# Shifts in age pattern, timing of childbearing and trend in fertility level across six regions of Nigeria: Nigeria Demographic and Health Surveys from 2003–2018

**DOI:** 10.1371/journal.pone.0279365

**Published:** 2023-01-20

**Authors:** Tubosun A. Olowolafe, Ayo S. Adebowale, Adeniyi F. Fagbamigbe, Obasanjo Afolabi Bolarinwa, Joshua O. Akinyemi

**Affiliations:** 1 Department of Epidemiology and Medical Statistics, Faculty of Public Health, University of Ibadan, Ibadan, Nigeria; 2 Department of Public Health, Lead City University, Ibadan, Nigeria; 3 Department of Public Health Medicine, University of KwaZulu-Natal, Durban, South Africa; 4 Department of Global Public Health, Canterbury Christ Church University, Canterbury, United Kingdom; University of Washington, UNITED STATES

## Abstract

**Background:**

Nigeria’s population is projected to increase from 200 million in 2019 to 450 million in 2050 if the fertility level remains at the current level. Thus, we examined the shifts in the age pattern of fertility, timing of childbearing and trend in fertility levels from 2003 and 2018 across six regions of Nigeria.

**Method:**

This study utilised the 2003, 2008, 2013, and 2018 Nigeria Demographic and Health Survey datasets. Each survey was a cross-sectional population-based design, and a two-stage cluster sampling technique was used to select women aged 15–49 years. The changes in the timing of childbearing were examined by calculating the corresponding mean ages at the birth of different birth orders for each birth order separately to adjust the Quantum effect for births. The Gompertz Relational Model was used to examine the age pattern of fertility and refined fertility level.

**Result:**

In Nigeria, it was observed that there was a minimal decline in mean children ever born (CEB) between 2003 and 2018 across all maternal age groups except aged 20–24 years. The pattern of mean CEB by the age of mothers was the same across the Nigeria regions except in North West. Nigeria’s mean number of CEB to women aged 40–49 in 2003, 2008, 2013 and 2018 surveys was 6.7, 6.6, 6.3 and 6.1, respectively. The mean age (years) at first birth marginally increased from 21.3 in 2003 to 22.5 in 2018. In 2003, the mean age at first birth was highest in South East (24.3) and lowest in North East (19.4); while South West had the highest (24.4) and both North East and North West had the lowest (20.2) in 2018. Similar age patterns of fertility existed between 2003 and 2018 across the regions. Nigeria’s estimated total fertility level for 2003, 2008, 2013 and 2018 was 6.1, 6.1, 5.9 and 5.7, respectively.

**Conclusion:**

The findings showed a reducing but slow fertility declines in Nigeria. The decline varied substantially across the regions. For a downward change in the level of fertility, policies that will constrict the spread of fertility distribution across the region in Nigeria must urgently be put in place.

## Background

The world population will increase from nearly 7.7 billion in 2019 to 8.5 billion in 2030 and further increase to about 9.7 billion by 2050 [1]. More than half of this rise is expected to come from sub-Saharan Africa (SSA) due to the high fertility that persists in the region [1]. Fertility decline in SSA has been considerably slower than the earlier declines observed in Asia, Latin America and the Caribbean, and Northern Africa at comparable stages of the fertility transition [2].

In SSA, countries such as South Africa, Ghana, Kenya, Gabon, and several others have progressed in the demographic transition process, but Nigeria is still struggling to lower its fertility level despite taking various measures to reduce it [3]. Nigeria is the most populous African nation and the seventh most populous country in the World [4]. Nigeria’s population grew from 56 million in 1963 to above 200 million in 2019, and it is projected to increase to 450 million in 2050 if nothing is done about its current fertility level [1]. The rapid growth of Nigeria’s population is facilitated by its high fertility rate. Therefore, if the world as a community wants to reduce the population growth rate and attain a replacement level in few years ahead, the fertility situation in Nigeria is important to reckon with.

The age pattern of fertility is essential in understanding the fertility situation in Nigeria because the likelihood of a woman having a child in a given time interval is strongly varied by age [5–7]. For instance, the mean number of children ever born (CEB) by the age of mothers’ ranges from 0.17 for 15–19 to 6.29 for 45–49 years (Table 1). To understand fertility levels and trends, the study of shifts in the age pattern of fertility and the timing of childbearing is critical [8]. The age pattern of fertility is the percentage contribution of the age-specific fertility rate (ASFR) to the total fertility rate (TFR). The ASFRs are used to examine the age patterns of the fertility of different populations over time because the fertility changes vary across the different age groups, and the contribution to TFR is highest in the prime reproductive age group. Usually, in a population with natural fertility, childbearing begins early though moderately and continues all through all the reproductive years, highest in the twenties and then declines to moderate levels for women in their forties [9].

**Table 1:** Mean number of children ever born by the age of mothers.

Age Group	North Central		North East		North West		South East		South-South		South West		Nigeria	
	2003	2018	2003	2018	2003	2018	2003	2018	2003	2018	2003	2018	2003	2018
	(μ±σ)	(μ±σ)	(μ±σ)	(μ±σ)	(μ±σ)	(μ±σ)	(μ±σ)	(μ±σ)	(μ±σ)	(μ±σ)	(μ±σ)	(μ±σ)	(μ±σ)	(μ±σ)
15–19	0.17±0.45	0.14±0.43	0.43±0.73	0.24±0.53	0.43±0.67	0.25±0.54	0.04±0.19	0.09±0.34	0.12±0.36	0.13±0.39	0.05±0.30	0.06±0.24	0.23±0.54	0.17±0.46
20–24	1.02±1.19	1.08±1.17	1.77±1.38	1.57±1.27	1.78±1.29	1.71±1.30	0.38±0.87	0.71±1.04	0.75±1.11	0.90±1.05	0.48±0.80	0.73±0.94	1.10±1.28	1.25±1.24
25–29	2.78±1.89	2.46±1.67	3.54±1.80	3.14±1.74	3.56±1.83	3.58±1.70	1.51±1.66	2.02±1.77	1.98±1.64	1.91±1.54	1.62±1.48	1.71±1.37	2.70±1.94	2.64±1.80
30–34	4±2.21	3.75±1.96	5.37±2.42	4.76±2.24	5.13±2.33	5.37±2.14	3.35±2.40	3.06±2.11	3.22±2.24	2.87±1.93	2.92±1.80	2.70±1.63	4.24±2.45	3.94±2.28
35–39	5.72±2.37	4.70±2.20	6.62±2.84	6.00±2.55	6.61±2.87	6.78±2.39	4.62±2.69	3.94±2.31	5.60±2.85	3.80±2.31	4.15±1.73	3.55±1.77	5.77±2.78	4.95±2.59
40–44	6.32±2.38	5.45±2.26	7.45±3.49	7.05±2.95	6.60±3.59	7.92±2.83	5.86±2.78	4.41±2.58	6.57±2.67	4.43±2.40	5.29±2.42	4.40±2.01	6.43±3.11	5.84±2.94
45–49	7.5±2.94	6.03±2.60	7.47±3.51	7.33±3.22	7.04±3.71	8.65±3.06	6.85±2.68	4.97±2.61	7.06±2.81	5.10±2.67	5.68±2.19	4.79±1.96	6.99±3.13	6.29±3.11

The knowledge about the effects of changes in the timing of fertility at all birth orders is essential for understanding the past and the future trajectories of reproductive behaviour [10]. Substantial changes in mean age at childbearing are often accompanied by a shift in the level of period fertility [11]. Thus, childbearing is influenced by period, age and lifetime fertility and duration since the last birth [10]. Fluctuation in fertility level occurs due to changes in the timing of fertility (tempo) and lifetime fertility of women (quantum) [12].

A similar shift in the age pattern of fertility was observed in many Asian countries and Latin America at the early stage of their fertility decline (UN DESA, 2002). Also, for fertility transition in Brazil, postponement in the second birth was identified without postponement in the first birth [10]. Likewise, Batyar [10] found a postponement of births, especially second births among reproductive women in Colombia [10]. The average age of women at their first birth in some European countries is about 30 years [5]. Although there is a clear difference between the age pattern of fertility in Africa and other regions, the shift in the age pattern of fertility has been observed in Africa was not different from what was previously observed in other regions of the world [13].

A study in Nigeria showed that the maternal age of a mother at first childbirth across all the regions in Nigeria ranged from 17.7 to 21 years and concluded that adolescent first birth is widespread in Nigeria [14]. This similarly perspective was highlighted by Singh et al. (2015) in a study conducted using mathematical curves to analyse fertility pattern [15]. In a recent study among out-of-school unmarried young women living in a metropolitan city in southwest Nigeria, Adebowale and colleagues (2019) found that the mean age at first sexual intercourse is 16.9 years [16].

Nigeria is a multi-ethnic country with diverse socio-cultural differences, so the indicators of fertility at the national level may be inadequate to explain childbearing behaviour in sub-population. It is essential to know if these indicators are the same across the regions of Nigeria. Although many works have focused on fertility in Nigeria; however, the age pattern of fertility and timing of childbearing has not been sufficiently considered in the analysis of fertility. Since changes in the level of fertility over time result from shifts in age patterns and timing of fertility, there is a need for a detailed assessment of the recent changes in the age pattern of fertility and the timing of childbearing. Therefore, this study aims to examine changes over the years in the age pattern of fertility, timing of childbearing and fertility levels in Nigeria as a whole and across its region.

## Methods and materials

### Study area

Nigeria has the largest population in Africa and the 14th largest in landmass. According to the 2006 Population and Housing Census conducted in Nigeria, the country’s population was 140,431,790 [17, 18], but the 2019 projection is based on the 2006 census figure, as the base year was above 200 million [19]. The country comprises 36 states with a Federal Capital Territory and is structured into six geopolitical zones, which are North Central (NC), North East (NE), North West (NW), South East (SE), South-South (SS) and South West (SW).

### Study design and data

The study utilized data from the 2003, 2008, 2013 and 2018 Demographic and Health Surveys (NDHS). Each survey was a cross-sectional population-based design, and a two-stage cluster sampling technique was used to select women aged 15–49 years. The 2003 NDHS programme made use of the sampling frame designed for the 1991 population census, while the sampling frame designed for the 2006 population and housing census was used for 2008, 2013 and 2018 NDHS but with modification due to expansion in the number of households between the census period and the survey years defined in all the survey rounds, the primary sampling unit (PSU) was a cluster tagged as the Enumeration Areas (EAs) from the 1991 and 2006 EA census sampling frames. Samples for the 2003 and 2008 surveys were selected using a stratified two-stage cluster design consisting of 365 clusters in 2003 NDHS and 888 clusters in 2008 NDHS. While 2013 and 2018 NDHS were conducted at three and two stages, respectively. For 2013, 893 localities were selected at the first stage with probability proportional to the size and with an independent selection from each sampling stratum. In the second stage, one EA was randomly selected from most of the selected localities. In a few larger localities, more than one EA was selected. In total, 904 EAs were selected. After selecting the EAs and before the main survey, a household listing operation was carried out in all the selected EAs. For 2018 NDHS, at the first stage, 1400 EAs were selected; and a household listing which served as a sampling frame was conducted on the selected EAs. In the second stage, 30 households were selected from each cluster by an equal probability of systematic sampling. The number of households interviewed in 2003, 2008, 2013, and 2018 was 7864, 34070, 40680 and 42000, respectively. The number of women aged 15–49 years interviewed for these year periods used in the study is given as 7620, 33385, 38948, and 41821, respectively. A detailed description of the methodology of the data set used for this study may be found in NDHS main report [15].

### Variable measurement

#### The timing of childbearing

The changes in the timing of childbearing were examined by calculating the corresponding mean ages at the birth of different birth orders of the study periods. The mean was calculated for each birth order separately to adjust the Quantum effect for births. The level of fertility is influenced by changes in the timing of childbearing (Tempo) and children ever born (Quantum) [12].

$$\mu =\frac{\sum_{i=I}^{I}x(i)f(i)}{\sum_{i=I}^{I}f(i)}$$ (1)

Where *μ* is the mean age of childbearing that measures the timing of childbearing; *x(i)* is the central age-point in the age interval, and *I* is the age group containing the upper age limit of the childbearing span; and *f(i)* denotes the fertility rate experienced by women in each age group.

#### Age pattern of fertility

The shifts in the age pattern of fertility can be examined by looking at observed or model age-specific fertility rates. However, due to reporting errors and the truncation effect; observed age-specific fertility may be inappropriate to describe the age pattern of fertility [20]. Thus, in an attempt to describe the fertility age pattern, several mathematical models have been proposed. These models have been used successfully to fit the age-specific fertility rates in different populations. One of such model was a relational method between a standard fertility schedule and any other schedule proposed by Brass [20]. The model is based on the assumption that the cumulative age pattern of fertility follows a Gompertz distribution function. However, it was found later that this model has two major shortcomings: first, it involves using total fertility (TF), which may be biased. The second shortcoming is the assumption that fertility has been constant. Nevertheless, Zaba’s Ratio method of 1981, which this study used, was an improved variant of the model proposed by Brass [21].

#### Data analysis

Two sets of data were used in the study. These are the women’s data set (individual recode) and children’s data set (child recode). All the variables needed in both the women’s data set and children’s data set for the study were extracted using SPSS. The variables used to estimate the numerator of TFR, the month and year of the child’s birth, were extracted from the children’s data set with the ID variable and matched with the women’s data. The observed ASFRs as presented in Fig 2 was obtained through direct estimation as described by Moultrie et al. [21]. While the estimated ASFRs were attained indirectly by following the procedures known as the “Ratio method” developed by Moultrie et al. [21]. The Gompertz parameters derived through these procedures were used to describe the age pattern of fertility and refine observed ASFRs. In the method, the average parities, _5_P_x_, of women in each age group (x, x+5) for x = 15, 20, ------- 45, were calculated. Then the fertility standard developed by Booth [20] was chosen to fit the model as follows:

$$\text{z}\left(\text{x}\right)–\text{e}\left(\text{x}\right)=\alpha +\beta \text{g}\left(\text{x}\right)+\text{c}/2{\left(\beta -1\right)}^{2}$$ (2)

$$\text{z}\left(\text{i}\right)–\text{e}\left(\text{i}\right)=\alpha +\beta \text{g}\left(\text{i}\right)+\text{c}/2{\left(\beta -1\right)}^{2}$$ (3)

Where:

$$z(x)=Y(x)=-\text{ln}(-\text{ln}(\frac{F(x)}{F(x+5)}))$$ (4)

$$g(x)=\frac{Y^{s}(x+5).\;\text{exp}(Y^{s}(x))+Y^{s}(x).\;\text{exp}(Y^{s}(x+5))}{\text{exp}(Y^{s}(x))-\text{exp}(Y^{s}(x+5))}$$ (5)

$$e(x)=gompit(\frac{Y^{s}(x)}{Y^{s}(x+5)})-g(x)$$ (6)

The plots of z(x)–e(x) against g(x) and z(i)–e(i) against g(i) (on the same set of axes) that were almost on the same line was used to fit the model. The values of α (intercept) and β (slope) are the parameters. The level of fertility (TFR) was estimated indirectly by applying the above-derived parameters (α & β) to the current fertility gompits. The parameters α & β for the periods were compared; α indicates the location of fertility, and β shows the spread in relation to the standard.

Sample weights were applied to each case to adjust for differences in the probability of selection. Weighting is important to increase the sample’s extent of representativeness and reduce the errors associated with sample selection bias.

### Ethical approval

Since the authors of this manuscript did not collect the data, we sought permission from the MEASURE DHS website and access to the data was provided after our intent for the request was assessed and approved on the 10th of March 2021. The DHS surveys are ethically accepted by the ORC Macro Inc. Ethics Committee and the Ethics Boards of partner organizations in different countries, such as the Ministries of Health. The women who were interviewed gave either written or verbal consent during each of the surveys.

## Results

The mean number of children ever born by the age of mothers across the Nigeria regions between 2003 and 2018 was presented in Table 1. In Nigeria, it was observed that there was a minimal decline in mean CEB between 2003 and 2018 across all the maternal age groups except aged 20–24 years. Based on the result, the pattern of mean CEB by the age of mothers was the same across the Nigeria regions except in North West. Between 2003 and 2018, the average CEB for women aged 30–34, 35–39, 40–44, and 45–49 increased from 6.61 to 6.78, 6.60 to 7.92, and 7.04 to 8.65, respectively. Table 2 shows Nigeria’s mean number of CEB to women aged 15–49 and 40–49 in 2003, 2008, 2013 and 2018 surveys. The variations in mean CEB were presented in the first four columns, and the last columns show the magnitude of change observed in the survey periods. CEB is a measure of cumulative fertility. In Nigeria, the mean number of CEB among women aged 15–49 was 3.0 in 2003 and upwardly shifted to 3.1 in 2008, while the mean CEB among women aged 40–49 fell from 6.7 to 6.1 within the 15-years period. Between 2008 and 2018, the mean CEB remain constant in Nigeria. In 2003, North East had the highest mean CEB of 3.9 per woman among women aged 15–49 but declined to 3.4 per woman in 2018, with an 11% decline between 2003–2018.

**Table 2:** Mean number of children ever born (CEB) to women aged 15–49 and 40–49 in Nigeria and across the region and percentage change in the year periods.

Mean number of CEB to women aged 15–49 in Nigeria and across the region					
	2003	2008	2013	2018	% Change
	(μ±σ)	(μ±σ)	(μ±σ)	(μ±σ)	(2003–2018)
Nigeria	3.02±3.2	3.1±3.1	3.1±3.0	3.05±3.0	1.0
North Central	2.99±3.1	2.9±2.9	2.6±2.6	2.78±2.7	-7.02
North East	3.88±3.4	3.9±3.3	3.7±3.3	3.44±3.2	-11.34
North West	3.69±3.3	4.0±3.3	4.0±3.4	3.94±3.5	6.78
South East	2.36±3.1	2.5±3.0	2.5±2.9	2.53±2.6	7.20
South South	2.49±3.1	2.5±2.8	2.4±2.7	2.44±2.5	-2.01
South West	2.02±2.4	2.3±2.4	2.4±2.4	2.34±2.2	15.84
**Mean number of CEB to women aged 40–49 in Nigeria and across the region**					
Nigeria	6.69±3.1	6.6±3.0	6.3±3.0	6.06±3.0	-9.42
North Central	6.98±2.8	6.4±2.7	5.6±2.4	5.73±2.5	-17.91
North East	7.46±3.5	7.4±3.3	7.3±3.3	7.17±3.1	-3.89
North West	6.78±3.6	7.7±3.2	7.8±3.1	8.27±3.0	21.98
South East	6.34±2.8	6.1±2.8	5.6±3.0	4.72±2.6	-25.55
South South	6.78±2.7	6.3±2.8	5.7±2.6	4.74±2.6	-30.09
South West	5.49±2.3	5.1±2.1	4.9±2.1	4.59±2.0	-16.39

Women of the South West region have consistently had the lowest fertility levels across all the survey years, though fertility stalled between 2003 and 2013 in the region. In North Central, a fertility decline was observed between 2003 and 2013 but stalled between 2013 and 2018. Between 2003 and 2018, while the mean CEB for women aged 15–49 only decreased from 2.5 to 2.4 in South-South, it increased from 2.4 to 2.5 in South East. Also, across the six geopolitical zones, fertility decline was observed among women aged 40–49 except in North West. The mean number of children born to women aged 40–49 in North West rose from 6.78 in 2003 to 8.27, about a 22% increase. The highest decrease in average cumulative fertility among women who had completed their fertility was found in SS (30%) and the lowest in NE (3%). At the national level, only a 9% fertility decline was observed among older women.

**Fig 1:**
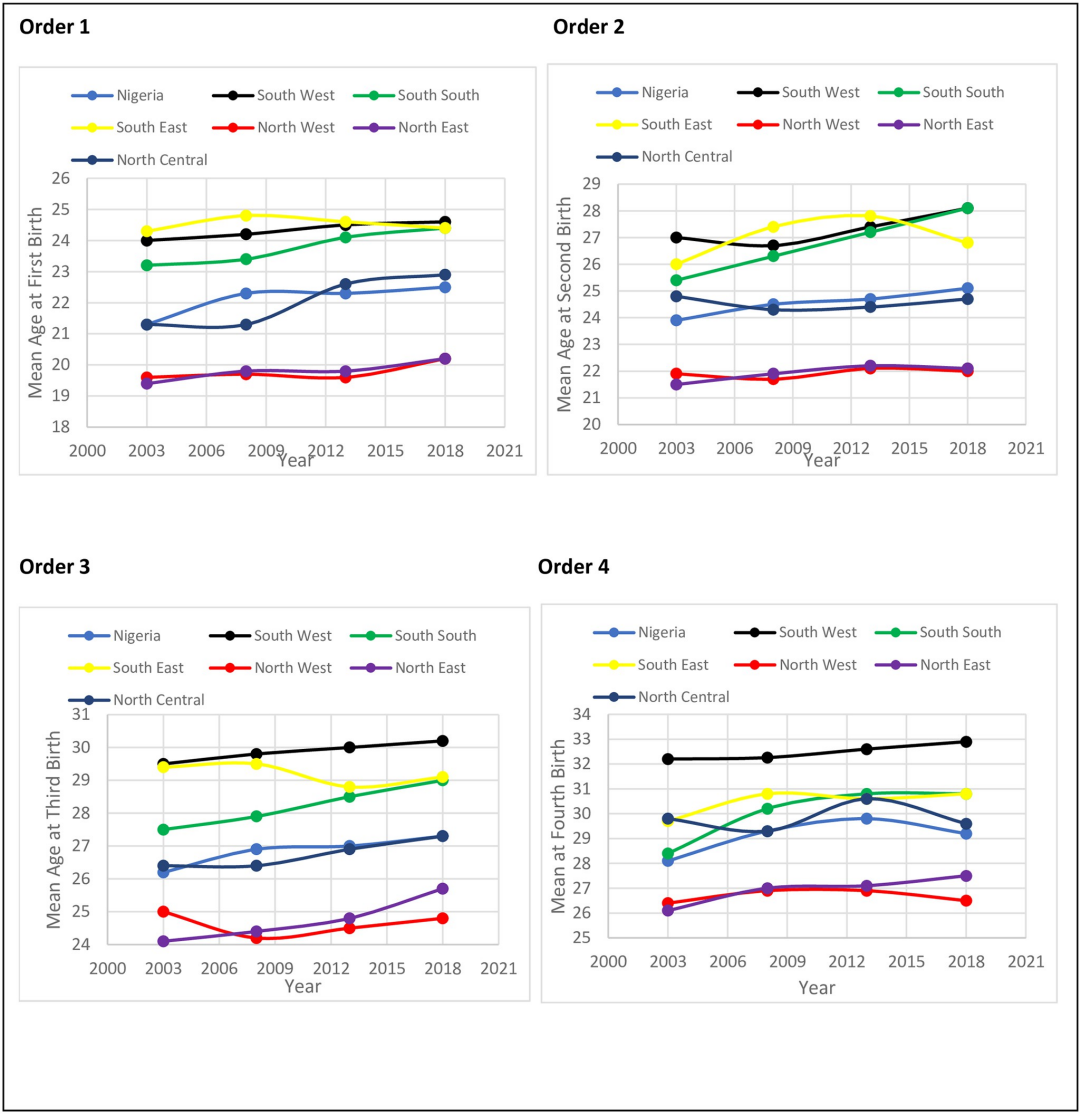
The mean age at childbearing (MAB).

Fig 1 depicts the mean age at childbearing at birth orders 1, 2, 3 and 4. The figure shows the trend in the timing of childbearing in Nigeria and the six geo-political zones. In the four birth orders, the mean ages at childbearing were lowest in North West and East. Nigeria’s mean age at first birth marginally increased between 2003 and 2018. This indicates that the timing of childbearing has not changed substantially in Nigeria. The mean age at first birth was highest in South East in 2003 and 2008 but the same in South West in 2013. In 2018, SW had the highest mean age at order 1. Across the regions, the mean age remained unchanged between 2003 and 2008. Whereas the mean age at first birth increased more in NC compared to other zones.

Nonetheless, there was at least a marginal increase in mean age at first birth order across the regions. The mean age at second birth marginally increased in Nigeria between 2003 and 2018. In NE, NW, and NC, the mean age at second birth was static. Meanwhile, a persistent increase in mean age at second birth was observed in SS, whereas, in SE, it rose between 2003 and 2013 but fell between 2013 and 2018. In SW, it increased between 2003 and 2018; but the rate of increase was minimal compared to that of SS.

In Nigeria, the mean age at third birth order increased between 2003 and 2008; but stalled between 2008 and 2018. The same pattern was observed in NC. Furthermore, the mean age of NE and NW were differentiated in the third birth order. An increase in mean age at third birth was observed in NE between 2003 and 2018, while in NW, the timing of childbearing at third birth order remains unchanged. In 2003, the mean age at the third birth order was almost the same in SW and SE; however, while there was an increase between 2003 and 2018 in SW, but it decreased between 2003 and 2018 in SE. Also, there was a steady increase in SS between 2003 and 2018. At birth order four, inconsistency was observed in the trend of the timing of childbearing in Nigeria, NC, SS, and SE. However, the mean age at fourth birth was clearly highest in SW, while NW and NE had the lowest. Between 2003 and 2018, the timing of childbearing did not change in Nigeria and across the regions except SW, where a marginal increase was observed.

**Fig 2:**
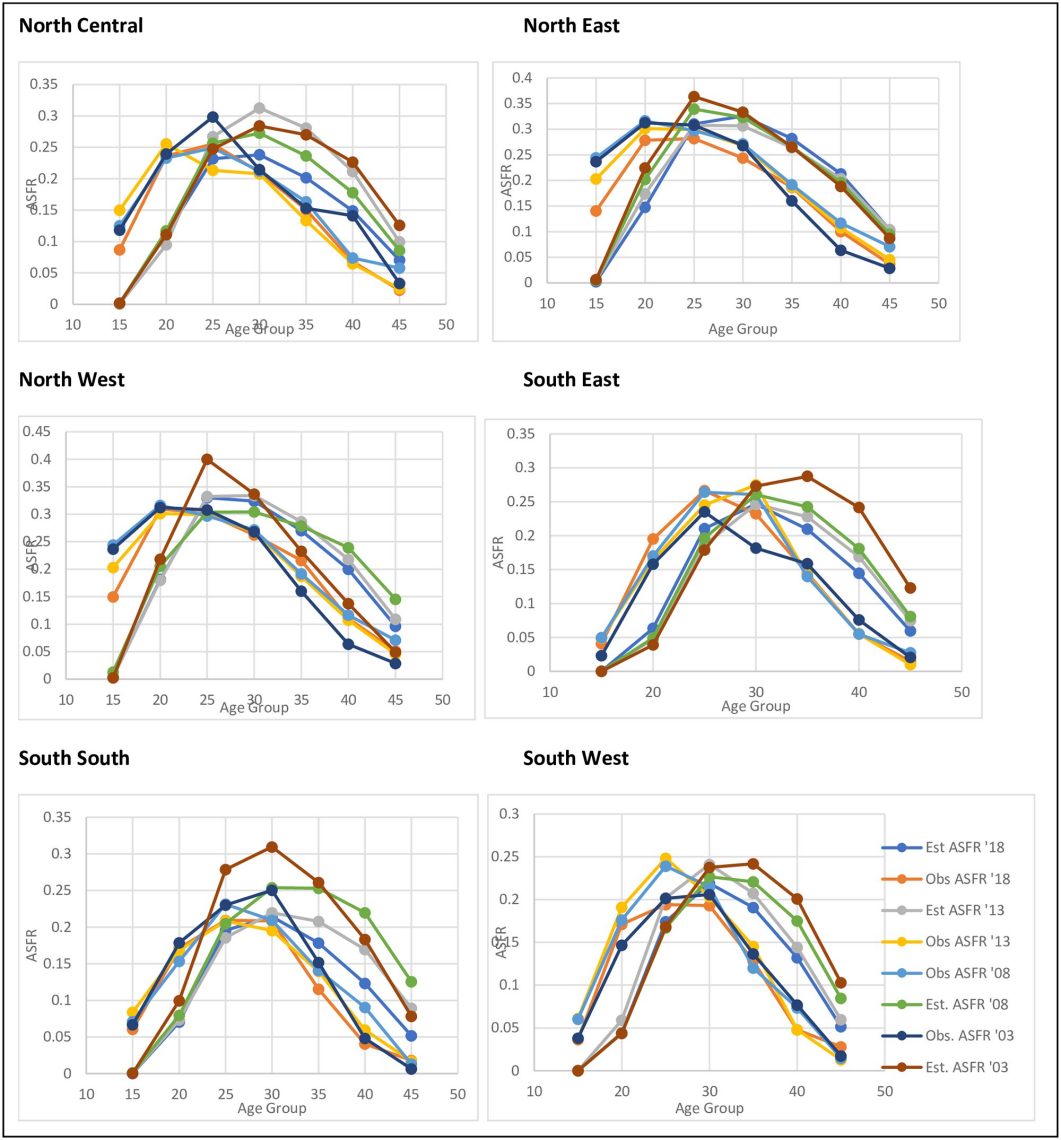
Age specific fertility rates observed and estimated by years, 2003–2018.

Fig 2 shows the Estimated and observed age-specific fertility rates by Age group for the six regions, 2003–2018. In NC, the estimated ASFRs curves reveal inconsistency in the shifts of age patterns in the periods. At younger ages, the curves of the estimated ASFRs for the year periods lie on each other; however, they began to be differentiated at age group 25–20, with estimated ASFRs 2018 as the lowest and followed by 2008. At age groups 30–34 and 35–39, 2003 curve was lower than the 2013 curve. Nevertheless, the curves of estimated ASFRs show that there was a shift in the age pattern of fertility at older ages in NC between 2003 and 2018. For NE, the curve of estimated ASFRs in periods 2003–2018 was similar, indicating there was no shift in the age pattern of fertility in the region between 2003 and 2018. The estimated ASFRs for the periods peaked at the age group 25–29 years. In NW, inconsistency in the shifts of age pattern of fertility was observed, with the estimated ASFRs for 2003 being the lowest at older ages.

According to the curve, the SE estimated ASFRs for 2018, 2013, 2008 and 2003 were almost the same at younger ages but varied at older ages. As expected, in later years, the 2003 estimated ASFRs were the highest, while that of 2013 and 2008 were closed. The estimated ASFRs for 2018 were the lowest. It shows there have been shifts in the age pattern of fertility in the SE between 2003 and 2018. For SS, the curve of the estimated ASFRs for the periods revealed inconsistency in the shifts of age pattern. For instance, the older ages ASFRs in 2013 were lower than that in 2008; in fact, that of 2003 was higher than in 2008. However, a shift in the age pattern of fertility was observed in SS between 2003 and 2018. Lastly, in SW, the curve of the estimated ASFRs for the periods differs at the older ages, with ASFRs in 2018 being the lowest and 2003 being the highest. This indicates a shift in the age pattern of fertility between 2003 and 2018 in SW.

The difference between the observed and estimated figures may be due to the quality of the data. Estimation of fertility indicators remains a challenge in many developing countries due to uncertainty about data quality [22]. Fertility data is of poor quality in Nigeria based on the evaluation of the quality of birth history data from 182 DHS surveys conducted in 69 countries show that fertility data tend to underestimate fertility in the country [23]. Fertility estimates in Nigeria rely heavily on information from surveys because of the incompleteness of the vital registration system and issues with censuses. The available survey data are prone to non-sampling and sampling errors, and it is almost impossible for such data to be without both content and coverage errors [24].

Table 3 below shows the estimates of the Gompertz parameters by Regions for the period 2003–2018. The results describe the patterns of fertility distribution among the six regions in Nigeria. The values of beta (β) of the result suggest two types of age patterns of fertility distribution among the regions. The beta values are measures of the pace of childbearing. A value greater than one depicts childbearing as concentrated in a narrow age, while a value less than one indicates the spread of the fertility distribution is wide. While Alpha values measure the location of childbearing. An increasing negative alpha value indicates a later onset and late end of childbearing [21]. It appears from the table that there is no shift in age patterns of fertility in Nigeria and the northern regions of Nigeria; however, in the southern regions, it appears that childbearing is concentrated in a narrow age.

**Table 3:** Gompertz Parameters Regions for the Period 2003–2018.

	2003	2008	2013	2018
**BETA VALUES**				
South West	0.9746	0.9479	1.0867	1.1036
South South	1.0601	0.8967	0.938	1.0703
South East	0.9857	1.0514	1.0555	1.0906
North East	1.1579	0.8324	0.9527	0.9738
North West	0.993	0.9669	0.9382	0.9730
North Central	0.9036	0.9808	1.0068	0.9897
Nigeria	0.9948	0.9398	0.9902	0.9958
**ALPHA VALUES**				
South West	-0.4704	-0.2449	-0.2919	-0.3071
South South	-0.1836	-0.3607	-0.3607	-0.1653
South East	-0.5393	-0.3806	-0.3729	-0.238
North East	0.1943	-0.0872	-0.0757	-0.1303
North West	0.067	0.0075	-0.0658	-0.0378
North Central	-0.2651	-0.1455	-0.2578	-0.1002
Nigeria	-0.1344	-0.1529	-0.1188	-0.1261

The beta values of SW, SE, and SS were greater than one in 2018, while they are less than one in other regions. Furthermore, the alpha values are negative for each of the survey years in all the regions, with the exception of NW and NE in 2003. The result depicts a relatively widespread fertility distribution in northern regions and national as well. Likewise, the alpha values indicate later onset and termination of childbearing in southern regions when compared with the northern region. Also, it appears from the Gompertz parameters that Nigeria has two distinct age patterns of fertility distribution. The values of β suggest similar age patterns of fertility exist between 2003 and 2018 in the northern regions and Nigeria; however, it appears that there have been shifts in the age pattern of fertility in southern regions.

**Fig 3:**
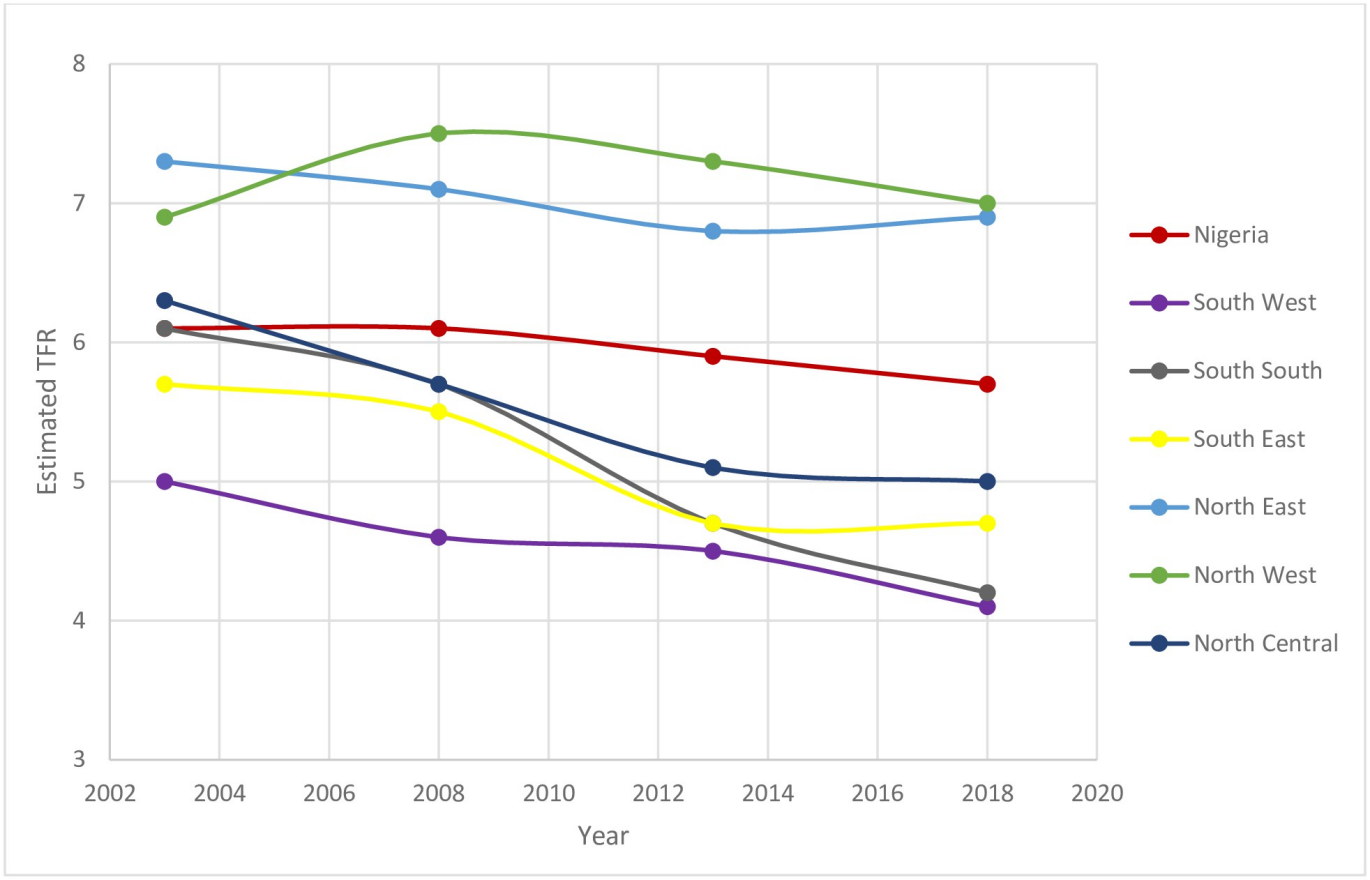
Trend in estimated total fertility rates by Nigeria and regions 2003–2018.

Also, the estimated total fertility rates of the model, as shown in Fig 3, reveal that there is a decline in fertility in all the regions, although the decline in NW and NE were marginal. Notably, the decline of fertility in the South-South (TFR declined from 6.1 in 2003 to 4.2 in 2018) was more rapid compared to other regions. The level of fertility has consistently been highest in NW and lowest in SW at any given time except in 2003, where the NE has the highest level of fertility. Fertility level remained the same between 2013 and 2018 in SE and increased by 0.1 in NE.

The S1 Appendix shows the fitting of current births and average parities on the same axis. As illustrated by the S1 Appendix of 2018 data, it appears that fertility is falling in Nigeria and across all the regions. Also, the diagnostic plots show parity omission and age exaggeration.

Fig 3 illustrates the trends in total fertility rates resulting from the application of the relational Gompertz model. The results, according to the figure, show that the SW had one of the more rapid declines in TFR and stagnation in fertility decline in the NE and NW.

In the SE, fertility decline was rapid between 2003 and 2013 but stalled between 2013 and 2018. Also, accelerated fertility decline was observed in SS and NC compared to other regions. Inconsistency was observed in NW. However, the level of fertility has been consistently lowest in the SW, and highest in the NW, except in 2003, where NE was the highest. The general trend is that regions with higher fertility had experienced a more gradual decline, while regions with low fertility had a more rapid decline, thus increasing the regional differentials in fertility.

## Discussion

Considering the demographic shifts and overall population growth, Nigeria will face many challenges in meeting basic demands, such as housing, education, and health care of the country. Nigeria is faced with a population explosion that is expected to push the population past 450 million people by the middle of the century if nothing is done about her fertility level. Since changes in the level of fertility over time result from shifts in age patterns and timing of fertility. Thus, this study described the shifts in the age pattern of fertility between 2003 and 2018 across six regions of Nigeria.

A shift in age patterns and timing of fertility is pertinent to changes in the level of fertility over time. A similar age pattern of fertility in Nigeria between 2003 and 2018 was established in this study. This pattern was also observed in all the regions in the northern part of Nigeria between 2003 and 2018. However, a marginal shift in the age pattern of fertility was found in the regions in the south regions (SE, SW, and SS). Age patterns of fertility between 2003 and 2018 indicate the early onset of childbearing in the Northern regions and the late termination of fertility in the Southern regions of Nigeria. This finding corroborates the outcome of previous studies conducted in Nigeria [14]. The difference in the socio-cultural landscape that can influence fertility in the northern and southern parts of Nigeria is a possible reason for North-South variability in the age pattern of fertility.

The age pattern of fertility where women continue childbearing as long as they are able such as in Nigeria is noted for high fertility. Fertility decline begins as fertility decreases at both early and later ages of reproductive years [25]. Across the globe, with more women gaining education and entering labour-force, there were delayed in entering motherhood. Most women in developed countries now have their children between the ages of 25–34 [5]. However, in Nigeria, childbearing begins early and continues throughout the reproductive years, highest in the twenties and then declines to moderate levels for women in their forties [9]. The analysis of this study demonstrated that the timing of childbearing has not really changed in Nigeria; thus, its effect on fertility level is negligible. The findings complement the outcome of existing research on fertility changes where changes in the timing of childbearing are expected before changes in fertility level [10].

In Nigeria, the total fertility rate estimated indirectly due to errors in birth reporting minimally declined from 6.1 in 2003 to 5.7 in 2018; the change of 0.4 recorded in our findings corroborates with the change documented in NDHS reports. However, our estimated TFR was inconsistent with that of NDHS reports, this may be due to errors associated with the surveys [26], which our model attempted to refine. The level of fertility was highest in North West and lowest in South West, and this pattern was consistent between 2003 and 2018. This finding is in line with earlier studies conducted in Nigeria [27]. The northern regions are dominated by people of Hausa/Fulani origin, mainly uneducated and predominantly Muslim. These groups have been marked as fertility drivers in Nigeria. This study further revealed that the decline of TFR was more rapid among women of the SS compared to the rest of the regions. This may not be unconnected with literacy level (ability to read and write), which was the highest in the region [15], and the decline of under-five mortality was more rapid in the region compared to other regions [28].

The values of Gompertz relation model parameters (α and β) used to provide a good-fit fall within the estimated the recommended ranges by Moultrie and colleagues [21]. Except for South East 2003 and South West 2003 data, all the regions in the year periods performed well. In Nigeria as a whole and across the six regions, the diagnostic plots of the GRM indicated that F-points lie above the P-points, which suggests there has been a fertility decline in the past. This finding is in agreement with a national study that shows a decline in fertility in Nigeria [15].

The postponement of first birth has not changed significantly, and the mean age at first birth obtained in this study echoes the slow pace of fertility transition in Nigeria. The mean age at first birth only increased slightly from 21.3 years in 2003 to 22.5 years in 2018, with the Southern and Northern regions having mean age at first birth of 24 years and 20 years, respectively. This finding agreed with the outcome of Fagbamigbe and Idemudia [29], which reported that early first childbirth has been prevalent in Nigeria [29]. Furthermore, the changing pattern of timing of childbearing in higher birth orders was not different from that of first birth. There is no evidence of an increase in the postponement of births in Nigeria [30]. Examining the effect of changes in the timing of childbearing on fertility level, the findings from this study confirm that changes in the timing of childbearing would result in changes in fertility level. In 2008, Tempo-adjusted TFRs were greater than the observed TFRs in first, second and third birth orders for all births combined in Nigeria. The difference observed suggests a decline in the timing of childbearing between 2003 and 2008. However, the observed TFRs were almost the same as Tempo-adjusted TFRs for all births combined in Nigeria for 2013 and 2018. The analysis demonstrated that the timing of childbearing has not changed in Nigeria; thus, its effect on fertility level is negligible. The findings complement the outcome of existing research on fertility changes where changes in the timing of childbearing are expected before changes in fertility level [10, 31].

As documented in the literature, several factors explain the persistence of high fertility in Nigeria. Early marriage and contraceptive use are among the main factors responsible for high fertility [32]. Findings have established the importance of contraceptive use and age at first sexual debut in facilitating fertility reduction [33–35]. In this study, a little change was observed in fertility reduction; this may be due to a marginal increase in the prevalence of contraceptive use across the regions [15]. Also, given the relatively early marriage that persists in the North West and North East [36], the fertility level remains above six. Considering the vital role of fertility in achieving sustainable development goals (SDGs), especially goals 1, 3 and 5, Nigeria will lag behind sub-Saharan African countries such as Ghana (TFR = 4.0), Kenya (TFR = 3.9), Gabon (TFR = 3.9), South Africa (TFR = 2.4) [2, 3], and others in achieving SDGs.

### Study limitations and strengths

The shifts in the age pattern of fertility and changes in the timing of childbearing are to be examined using observed age-specific fertility rates over time. Due to the incompleteness of the vital registration system in Nigeria, observed age-specific fertility may not be suitable for this study. However, a model designed to circumvent this problem was used in this study; and it was used successfully to fit the age-specific fertility rates in different populations. The strength of this study is the use of large national representative cross-sectional datasets across 15 years conducted in Nigeria to estimate future fertility level trajectories.

### Implications for policy and research

The study has several implications for policy and research in Nigeria. Fertility level estimates will inform the choice of policy that will be recommended and also make specific group policies to avert the danger of unplanned fertility in the country. The study also informed research by estimating one of the vital components of population studies and presenting new findings that future studies can reference.

## Conclusion and recommendations

The indirect techniques and mathematical models adopted in this study provided plausible estimates of fertility levels and clearly described the future trajectory of fertility in Nigeria. The similarity of a relatively widespread fertility distribution exists in Nigeria as an entity and Northern regions. There have not been substantial shifts in the age patterns of fertility between 2003 and 2018 in Nigeria. Slight shifts in age patterns of fertility were observed in Southern regions, but in the core North of the country, there were no shifts. Also, the study demonstrated that the timing of childbearing has not changed in Nigeria; this is evident in the change in the fertility level observed in the country. This study has also revealed that fertility levels are still high in Nigeria. There were regional differentials in fertility levels and trends. Notably, Southern regions are moving to the point of transiting to the second phase of transition, that is where TFR equals 4.0, while fertility levels are above 6 children per woman in NW and NE. The level of fertility in Nigeria, as indicated by TFR, implies that, if the current trend persists, Nigeria is faced with a population explosion that is expected to make its population size above 450 million people by 2050. To avoid the crises that may arise due to population explosion due to high fertility levels and population growth, immediate actions to reduce fertility drastically should be put in place. With a consistent and rapid fertility decline, Nigeria will have fewer children to cater for; and a larger proportion will be in the economically productive age bracket. This is the demographic dividend that Nigeria can benefit from if something urgent is done about its fertility level. For a downward change in the level of fertility, policies that will constrict the spread of fertility distribution across the region in Nigeria must urgently be put in place.

## Supporting information

S1 Appendix (DOCX)
