# Supplementary material for: Shifts in age pattern, timing of childbearing and trend in fertility level across six regions of Nigeria: Nigeria Demographic and Health Surveys from 2003–2018
Source: PLoS One. 2023 Jan 20;18(1):e0279365. doi: 10.1371/journal.pone.0279365 (PMC9858060; doi:10.1371/journal.pone.0279365)
Supplement: S1 Appendix — (DOCX) [file pone.0279365.s001.docx]

**Appendix**

App. 1: Diagnostic plots of Nigeria 2018 data App. 2: Diagnostic plots of Nigeria 2013 data

App 3: Diagnostic plots of Nigeria 2008 data App 4: Diagnostic plots of Nigeria 2003 data

App 5: Diagnostic plots of South West 2018 data App 6: Diagnostic plots of South West 2013 data

App 7: Diagnostic plots of South West 2008 data App 8: Diagnostic plots of South West 2003 data

App. 9: Diagnostic plots of South South 2018 data App 10: Diagnostic plots of South South 2013 data

App 11: Diagnostic plots of South South 2008 data App 12: Diagnostic plots of South South 2003 data

App 13: South East Diagnostic plots of 2018 data App 14: South East Diagnostic plots of 2013 data

App 15: South East Diagnostic plots of 2008 data App 16: Diagnostic plots of South East 2003 data

App 17: Diagnostic plots of South East 2003 data App18: Diagnostic plots of North West 2013 data

App 19: Diagnostic plots of North West 2008 data App 20: Diagnostic plots of North West 2003 data

App 21: Diagnostic plots of North East 2018data App 22: Diagnostic plots of North East 2013data

App 23: Diagnostic plots of North East 2008data App 24: Diagnostic plots of North East 2003data

App 25: Diagnostic plots of North Central 2018 data App 26: Diagnostic plots of North Central 2013 data

App 27: Diagnostic plots of North Central 2008 data App 28: Diagnostic plots of North Central 2003 data
